# The mediating effect of alexithymia in the symptom burden and depression in patients with maintenance hemodialysis

**DOI:** 10.3389/fpsyg.2025.1570190

**Published:** 2025-07-08

**Authors:** Jing Liu, Ning-ning Xia, Hongying Wang, Yinying Yuan, Leijuan Xiao, Anne Xiang

**Affiliations:** Department of Nephrology, Nanjing BenQ Medical Center, The Affiliated BenQ Hospital of Nanjing Medical University, Nanjing, Jiangsu, China

**Keywords:** maintenance haemodialysis, alexithymia, depression, symptom burden, mediating effect

## Abstract

**Aim:**

This study aimed to investigate the prevalence of alexithymia among patients receiving maintenance haemodialysis (MHD) and whether it plays a role in the relationship between symptom burden and depression in this population.

**Background:**

The prevalence of depression among patients undergoing MHD is increasing. Numerous studies have found strong links between alexithymia, symptom burden, and the development of depression in this population. However, the underlying mechanisms and alexithymia's specific mediating role in the relationship between symptom burden and depression are poorly understood and have received little attention in the existing literature.

**Methods:**

This study included 380 MHD patients in a haemodialysis center, with a mean age of 58.98 ± 13.86 years, using a self-designed patient general information questionnaire, disease-related information questionnaire, dialysis patient symptom burden scale, depression scale, and Toronto Alexithymia Scale (TAS-20). A regression model of the factors influencing depression was developed using structural equation modeling.

**Results:**

MHD patients had a DFSSBI score of 77.41 ± 45.74, a TAS-20 score of 55.36 ± 11.17, and a Patient Health Questionnaire (PHQ-9) score of 6.07 ± 4.60. The burden of symptoms was positively correlated with alexithymia and depression (*r* = 0.367, 0.776, *P* = 0.000). The regression model had a high goodness of fit (χ^2^/df = 1.604, RMSEA = 0.040, GFI = 0.986, CFI = 0.999, TLI =0.997). The structural equation model analysis found the following: symptom burden was a positive predictor of alexithymia, β = 0.296, *P* < 0.001; alexithymia was a positive predictor of depression, β = 0.752, *P* < 0.001; and symptom burden was a positive predictor of depression, β = 0.141, *P* < 0.001.

**Conclusion:**

The level of depression in MHD patients is closely related to the burden of symptoms and alexithymia, with alexithymia serving as a partial intermediary between the two. Addressing the emotional wellbeing and symptom load of MHD patients is critical to relieving their depressive symptoms.

## 1 Introduction

The global prevalence of kidney diseases has increased in recent years. Chronic kidney disease affects ~10% of the global population. According to statistics, End-Stage Renal Disease (ESRD) will affect ~14.5 million people worldwide by 2030 (Bharati and Jha, 2022). Maintenance haemodialysis (MHD) is the primary intervention for people with end-stage renal disease (ESRD), and it has been demonstrated to be safe and therapeutically effective. The 2020 Report on Kidney Diseases from China revealed that the prevalence rate of MHD in the country is 402.18 per million people (Zhang et al., 2020).

MHD therapies may extend patients' lives. However, the need for prolonged dialysis introduces significant psychosocial challenges and symptomatological burdens, which can severely impair their quality of life (Chen et al., 2017). Patients with MHD are at high risk of developing emotional disturbances as a result of the illness's burden, including time constraints, dietary restrictions, functional limitations, changes in self-perception, and fear of death (Pojatić et al., 2021). Depressive symptoms are associated with the prognosis of various diseases, as well as mortality rates in both clinical and general populations. There is also an association between depression and increased mortality in patients with MHD (Meng et al., 2022; Wang et al., 2023).

In China, nearly 55.1% (Ye et al., 2022; Meng et al., 2022) of individuals receiving MHD suffer from depression, which has a significant impact on their own and their families' personal lives. Dialysis can cause various depressive symptoms, including itchy skin and skin plaques (van der Willik et al., 2022), sexual dysfunction (Keskin et al., 2019), behavioral deficits, sleep deprivation (Lindner et al., 2015), unemployment problems, inability to communicate effectively, and lack of control over things (Bargiel-Matusiewicz et al., 2010). All of the above factors contribute to severe stress, and people with MHD must rely on their own internal mental abilities to cope. Due to the high level of stressors and increased frustration in MHD patients, these patients begin to react with immature self-defense mechanisms such as denial (which is the most common expression) (Fukunishi et al., 1992b). Certain studies have suggested that avoiding stress causes alexithymia (Besharat and Shahidi, 2011). MHD is a lengthy and lifelong treatment. Therefore, the patient's psychological factors are critical to its continuity and effectiveness. Patient has low confidence, which may harm treatment consistency. Thus, it is necessary to assess internal cognition for MHD patients (Ramya and Jagadeswaran, 2024).

Alexithymia has been identified as an intrinsic factor in the development of psychosomatic disorders (Sifneos, 1996). It refers to deficits in cognitive processing, regulation, and expression of emotions, which result in difficulties identifying and describing one's own emotional experiences and an inability to express emotions appropriately (Lyvers et al., 2017).

As research progressed, alexithymia became increasingly linked to physical illnesses (López-Muñoz and Pérez-Fernández, 2020) and chronic conditions (Fukunishi et al., 1992a), such as hypertension (Di Giuseppe and Conversano, 2022), diabetes, and asthma (Ricciardi et al., 2023). It has provided a new understanding of alexithymia, focusing on deficits in interoceptive awareness and emotion regulation, especially in recognizing and articulating emotional states. Some researchers have proposed that alexithymia may impair treatment compliance, influence treatment outcomes in clinical settings, and increase mortality risk in the general population (Peters et al., 2015). Recently, it was found that alexithymia was independently associated with the presence of depression and its progression in haemodialysis patients (Sifneos, 1996). However, it is largely unknown whether alexithymia influences long-term prognosis in this population, the underlying mechanisms, and its specific role in mediating the relationship between symptom burden and depression in MHD patients. Therefore, this study analyzed the pathway relationship between symptom burden, alexithymia, and depression in MHD patients in order to reduce depression levels, promote disease regression, and improve patient survival quality.

## 2 Methods

### 2.1 Ethical statement

The study complies with the Declaration of Helsinki. Ethical approval was obtained from the institutional Ethics Committee at BenQ Hospital Affiliated to Nanjing Medical University, with approval number [2022-KL010]. All study participants provided informed consent.

### 2.2 Study design and population

The study design is observational and cross-sectional. We used G^*^Power 3.1.9.7 software to determine the sample size for the study, with *f*^2^ = 0.15, power = 0.95, and α = 0.05, indicating a minimum of 107 cases. The inclusion criteria were as follows: ① age ≥18 years old; ② MHD ≥3 months; ③ normal cognitive function; and ④ informed consent and voluntary participation. Exclusion criteria included: ① individuals with language and auditory impairments; ② patients with end-stage renal disease resulting from acute kidney injury or other malignancies; and ③ individuals diagnosed with Alzheimer's disease. Researchers collected data from 392 patients receiving treatment at a haemodialysis facility within BenQ Hospital in Nanjing. The study was conducted from March to May 2022.

### 2.3 Measures and data collection

Following a standardized training session, the research team handed out questionnaires and collected them on the spot. The researchers first explained the study's purpose to the participants before conducting an in-person survey once they had obtained informed consent. In addition, they removed incomplete questionnaires with invalid responses by individually verifying their completeness. They used two-person input to ensure the data's accuracy. Four of the 392 distributed questionnaires could not complete the survey due to renal transplantation, and eight chose not to participate, resulting in 12 incomplete entries. Finally, 380 people participated in the survey, resulting in a remarkable recovery rate of 96.9%.

### 2.4 General data questionnaire

A general data questionnaire was developed by the research team, encompassing both demographic and health-related variables. Demographic variables included sex, age group, relationship status, employment status, monthly income, educational background, and payment method. Health-related variables included primary illness, dialysis duration, serum calcium and phosphorus levels, parathyroid hormone (PTH) levels, *Kt*/*V* values, and the use of antidepressant medications.

### 2.5 Dialysis Frequency Severity and Symptom Burden Index (DFSSBI)

The symptom burden in MHD patients was assessed using a scale developed by Danquah et al. (2010), which was adapted from the original Dialysis Symptom Index (DSI) (Weisbord et al., 2007). This instrument assesses the number of symptoms reported by dialysis patients and their frequency, severity, and level of distress. The scale consists of 30 items, with 25 physical and five psychological symptoms. Symptom occurrence is recorded dichotomously (0 = no, 1 = yes), whereas symptom frequency and severity are rated on a four-point Likert scale (1 = occasionally/mild, 2 = sometimes/moderate, 3 = often/severe, 4 = always/very severe). Symptom-related distress was measured on a five-point Likert scale (0 = not at all, 1 = mild, 2 = moderate, 3 = severe, 4 = very severe). The total score ranges from 0 to 360, with higher scores indicating a more severe symptom burden. The scale is reliable and valid in international studies, with Cronbach's α of 0.923 and a content validity index (CVI) of 0.939. Zhou et al. (2013) used the Chinese version of the Dialysis Symptom Burden Index (DFSSBI) in a study of 64 MHD patients to investigate the relationship between symptom burden and quality of life. The Chinese version had a Cronbach's α of 0.89 and a test-retest reliability of 0.91, indicating its suitability for assessing symptom burden among MHD patients.

### 2.6 Patient Health Questionnaire (PHQ-9)

The Patient Health Questionnaire (PHQ-9) (Tian et al., 2021) instrument was used to assess depressive symptoms. This assessment tool, which is based on the criteria established in the Diagnostic and Statistical Manual of Mental Disorders (DSM-IV), provides a clear and effective means of self-evaluating depression. Equally important, this self-assessment scale for depression screening is simple to use, effective, and does not require the involvement of professionals. Participants complete the scale based on their experiences from the previous 2 weeks. The scale contains nine items and provides four response options for each: almost every day, more than 1 week, several days, and not at all. The scoring system assigns values ranging from 0 to 3, resulting in a maximum cumulative score of 27 points. An increase in scores is associated with an increased risk of developing depression. This scale has a Cronbach's α of 0.839 (Gupta et al., 2018). This study defined a score of 0 to 4 as the absence of depression, whereas a score of 5 or higher indicated the presence of depression. The scale has a Cronbach's α coefficient of 0.836, indicating reliability and widespread use in assessing depression in China.

### 2.7 Toronto Alexithymia Scale (TAS-20)

Toronto Alexithymia Scale (TAS-20) was evaluated using Taylor et al.'s revised TAS-20 scale, which was translated by Yuan et al. (2003) and included 20 items in three dimensions: affect recognition disorder (seven items), affect description disorder (five items), and extroverted thinking (eight items). This scale is frequently used to assess the quality of life in China. Each entry was scored on a five-point Likert scale, with 1–5 representing “completely disagree” to “completely agree,” and individual entries were reverse-scored, yielding a cumulative score range of 20–100 points, which was positively correlated with the severity of the affective disorder. The Cronbach's α coefficients for all dimensions were ≥0.800.

### 2.8 Statistical analysis

The analysis involved two people entering data into Microsoft Excel 2016 and examining the results with SPSS 26.0. Categorical data were summarized using frequency counts and percentage calculations. Researchers presented measurement data as mean ± standard deviation or median with quartile values.

A single-factor analysis with a non-parametric approach was used to evaluate data that did not meet the normal distribution criteria. Correlation analysis was performed using the Spearman correlation coefficient, and mediating effects were investigated using structural equation modeling with AMOS 26 software. Furthermore, researchers repeated the bootstrap procedure 5,000 times. A mediation effect was confirmed when the 95% confidence interval, adjusted for deviation, excluded 0. A *P*-value < 0.05 was considered statistically significant.

## 3 Results

Table 1 depicts the characteristics of the population under study. The analysis revealed that 222 out of 380 patients had depressive symptoms, representing a 58.4% incidence. A total of 158 people, or 41.6% of the sample, reported no depressive symptoms. According to the findings, 49 cases (12.9%) were diagnosed with moderate depression, while 153 cases (40.3%) presented with mild depressive symptoms. Then, six people, or 1.6% of the sample, suffered from severe depression, while 14 people, or 3.7% of the sample, experienced moderate to severe depression. The average symptom burden score was 77.41, with a standard deviation of 45.74, representing 13.11 symptoms ± 6.01. Additionally, the distress scores were 25.22 ± 15.42, severity scores 25.43 ± 15.29, and frequency scores 26.71 ± 15.31. Alexithymia scores were 55.36 ± 11.17, affect recognition disorder scores were 20.12 ± 5.27, affect description disorder scores were 13.43 ± 3.42, and extroverted thinking scores were 21.57 ± 3.23.

**Table 1:** Characteristics of the study population (n = 380).

**Variable**	**Mean ±SD**	***N* (%)**
Age (years)	58.98 ± 13.86	
Female gender		132 (34.74)
Without a spouse		95 (25.00)
Unemployed		298 (78.42)
**Education**		
Middle school and below		165 (43.42)
Technical secondary school and senior high school		112 (29.47)
Junior college and above		103 (27.11)
**Income**		
≤5,000 yuan		96 (25.26)
5,000–10,000 yuan		154 (40.53)
>10,000 yuan		130 (34.21)
**Medical expenses payment methods**		
Self-paid		14 (3.68)
Insured for urban residents		321 (84.47)
New rural cooperative medical care system		45 (11.84)
**Dialysis age**		
<5 years		234 (61.58)
5–10 years		103 (27.11)
>10 years		43 (11.32)
**Primary diseases**		
Hypertensive nephropathy		103 (27.11)
Diabetic nephropathy		97 (25.53)
Glomerulonephritis		96 (25.26)
IGA nephropathy		3 (0.79)
Polycystic kidney disease		15 (3.95)
Drug-induced kidney damage		1 (0.26)
Other cases		65 (17.11)
Blood calcium (mmol/L)	1.80 ± 0.60	
Blood phosphorus (mmol/L)	2.07 ± 0.74	
PTH (pg/ml)	217.79 ± 150.35	
Kt/V	1.51 ± 0.27	
Antidepressants drug use		11 (2.89)

SD, standard deviation.

Table 2 shows a positive correlation by Pearson's correlation analysis (*P* < 0.01) between symptom burden, alexithymia, and total depression scores among MHD patients.

**Table 2:** Correlation analysis between symptom burden, depression, and alexithymia in MHD patients (*n* = 380, *r*-value).

**Variable**	**1**	**2**	**3**	**4**	**5**	**6**	**7**	**8**	**9**
1 Total depression scores	0.367^**^								
2 Total symptom burden scores	0.370^**^	1							
3 Frequency	0.364^**^	0.992^**^	1						
4 Severity scores	0.362^**^	0.997^**^	0.983^**^	1					
5 Distress scores	0.776^**^	0.997^**^	0.982^**^	0.998^**^	1				
6 Total Alexithymia scores	0.765^**^	0.294^**^	0.292^**^	0.293^**^	0.290^**^	1			
7 Affect recognition disorder scores	0.772^**^	0.281^**^	0.280^**^	0.280^**^	0.277^**^	0.959^**^	1		
8 Affect description disordert scores	0.613^**^	0.289^**^	0.292^**^	0.287^**^	0.285^**^	0.964^**^	0.913^**^	1	
9 Extroverted thinking scores	0.367^**^	0.249^**^	0.242^**^	0.251^**^	0.249^**^	0.866^**^	0.714^**^	0.778^**^	1

** *P* < 0.01.

**Figure 1:**
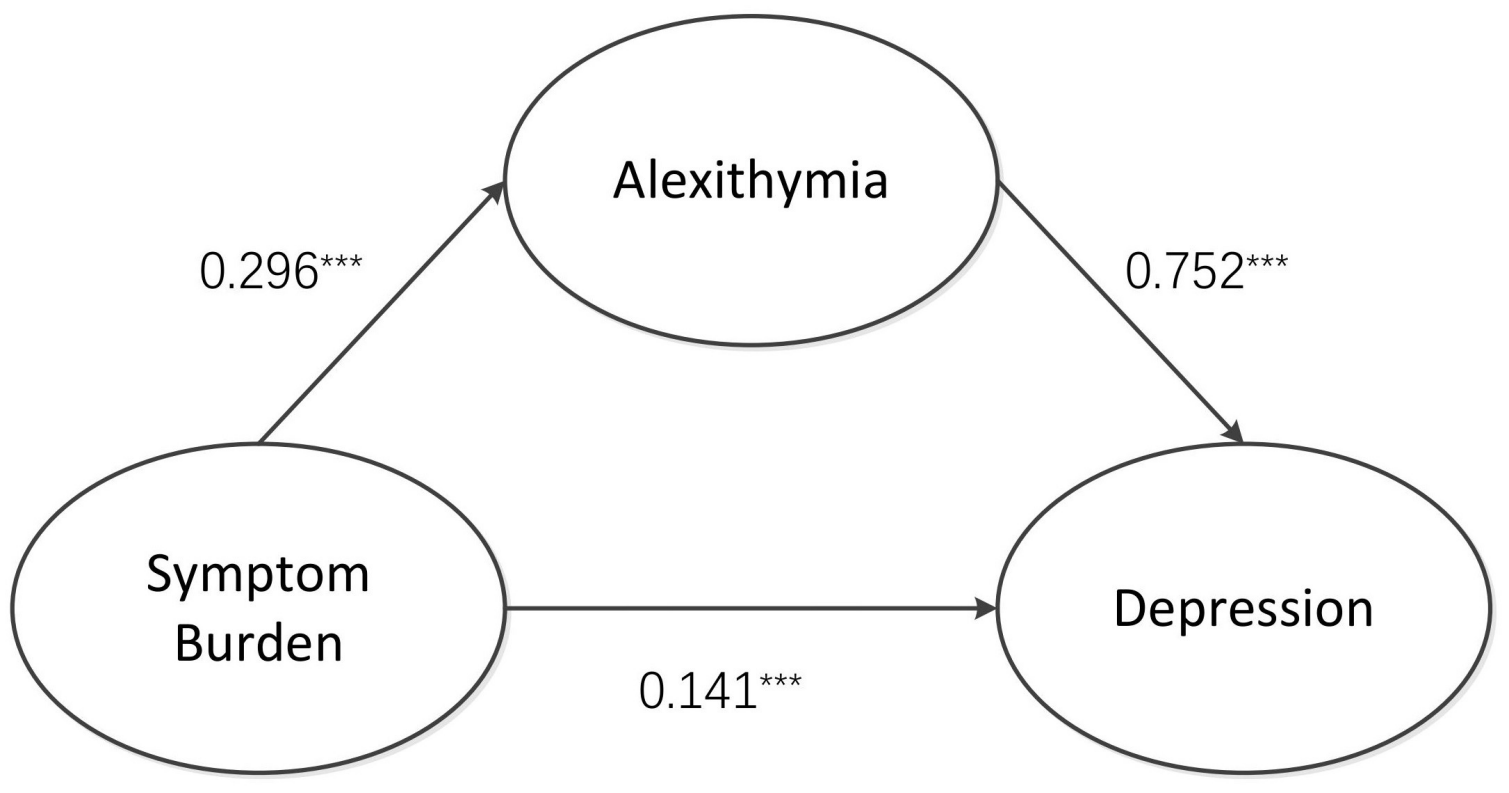
Mediating effects of symptom burden, depression, and alexithymia in MHD patients.

Based on expert insights and correlation analysis results, it is proposed that the burden of symptoms influences depression in two ways: directly on depression itself and indirectly mediated by dysphoria. The study used a structural equation model to chart the associations between symptom burden, depression, and alexithymia, emphasizing alexithymia's mediating role in the relationship between symptom burden and depression. The maximum likelihood method (ML) was used to evaluate model parameters. The fitting results are as follows: χ^2^*/df* = 1.604 (1 < χ^2^*/df* < 5), RMSEA = 0.040 (<0.10), GFI = 0.986 (>0.90), CFI = 0.999 (>0.90), and TLI = 0.997 (>0.90). As shown in Figure 1 and Table 3, the model fit met the standard, indicating that the model was better suited to the data. The path coefficient results show that symptom burden is a positive predictor of alexithymia, β = 0.296, *P* < 0.001. Alexithymia was a positive predictor of depression, β = 0.752, *P* < 0.001, and symptom burden was a positive predictor of depression, β = 0.141, *P* < 0.001.

**Table 3:** Path analysis of factors influencing the depression of MHD patients.

**Path**	**B**	**β**	**SE**	**t**	***P-*value**
Symptom burden → Alexithymia	0.049	0.296	0.009	5.769	<0.001
Alexithymia → Depression	1.359	0.752	0.081	16.813	<0.001
symptom burden → Depression	0.042	0.141	0.010	4.318	<0.001

SE, standard error.

According to the mediating effect analysis (Table 4), alexithymia played a significant mediating role in the relationship between depression and symptom burden. The value of this mediating effect was 0.22 [95% confidence interval of (0.150, 0.293)] and did not include 0. This finding indicated that the effect of symptom burden had been established.

**Table 4:** Mediating effects analysis table of symptom burden, depression, and quality of life in MHD patients.

**Mediating path**	**Effect value**	**SE**	**LLCI**	**ULCI**
Symptom burden → Alexithymia → depression	0.223	0.037	0.150	0.293
Direct effect	0.141	0.041	0.064	0.224
Total effect	0.364	0.055	0.260	0.476

SE, standard error; LLCI, the lower limit confidence interval; ULCI, the upper limit confidence interval.

## 4 Discussion

This study found that MHD patients had a higher symptom burden score (77.41 ± 45.74) than reported by Wang et al. (2017) Patients with MHD had reduced glomerular filtration function, and the residual renal units were unable to excrete metabolites efficiently, resulting in toxins accumulating in the body and causing a variety of symptoms. The top 10 symptoms in the incidence of symptom burden in this study population included, in order, dry mouth, itching, dry skin, fatigue, dizziness, worry, difficulty sleeping, easy to wake up, anxiety, and irritability, which were consistent with the findings of domestic and international studies (Flythe et al., 2015). In recent years, more and more studies have found that patients with MHD are troubled by more than one symptom, which tends to co-occur and interact with one another, aggravating the patient burden, reducing treatment adherence, and seriously affecting the patients' organismic functioning and quality of life (Zhou et al., 2023).

The results of this study revealed that the incidence of alexithymia in MHD patients was 60.3%, which was significantly higher than the 29.3% observed in elderly patients with chronic diseases in general (Fenglin and Jianping, 2018) and comparable to the incidence of alexithymia in cancer patients (Tang et al., 2021). The prevalence in this study is also significantly higher than the 13.9% reported by Kojima et al. (2010) in a cohort of 230 haemodialysis patients. Participants in current study had a higher mean age (58.98 ± 13.86 vs. 56.0 ± 9.6 years) and longer dialysis vintage (4.75 ± 4.52 vs. 4.5 ± 1.2 years). This could explain the discrepancy in results. Several studies found that alexithymia was more common in HD patients over the age of 60 and in HD patients over the age of 65 whose treatment lasted longer than in HD patients of the same age whose haemodialysis treatment had only recently begun (Onor et al., 2010). In this study, the content scores for emotional expression disorders ranged from high to low, including emotion recognition disorder, extroverted thinking, and emotion expression disorder. 30%−60% of patients with MHD have cognitive deficits (Griva et al., 2010), and patients have difficulty in perceiving facial emotions in others. This difficulty may lead to misinterpretation of social signals, impairing emotional recognition in such patients. MHD patients must be treated 2–3 times weekly for 3.5–4.5 h each (Zhang et al., 2020). Treatment cycles are long and as a result most patients are unable to work and have a low and inefficient ability to solve problems in their lives. They face discrimination and have low self-esteem, and their refusal to communicate with the outside world may lead to extroversion disorder in this population. Studies (Wang et al., 2022) have shown that the lower the patient's level of education, the more severe the narrative disorder. In this study, only 27.1% of the patients had a higher education or higher, and those with less education may have a limited vocabulary to accurately describe emotions, leading to impaired emotional expression. The prevalence of depression among MHD patients in this study was 58.4%, which was lower than the findings of Pretto et al. (2020) but still higher than the general population, most likely due to the use of different assessment tools. MHD is a standard treatment for end-stage renal disease, requiring dialysis 2–3 times per week for 3.5–5.5 h each time. The treatment cycle is long and lasts a lifetime. Long-term dialysis has an impact on patients' occupational and family life, making them more prone to negative emotions such as depression than the non-dialysis population.

There was a significant positive correlation between symptom burden, alexithymia, and depression. A study (Khosravani et al., 2020) found a direct link between depression and affective disorders. Specifically, the more severe the symptom burden, the lower the ability to narrate emotions and the greater the level of depression. The analysis could be attributed to alexithymia being an immature defense mechanism against physical stressors (Donges and Suslow, 2017). Previous research has shown that HD patients are more likely to experience somatisation and projection than the general population. Somatisation is an immature defense mechanism in which frustration-induced discomfort manifests as somatic symptoms, whereas projection occurs when HD patients attribute their unacceptable thoughts and desires to others (Carvalho et al., 2013). Given that immature defenses have been linked to high levels of alexithymia on both specific clinical samples and the general population, it is clear that alexithymia is a construct that can impede communication with medical staff in various ways (Helmes et al., 2008). During treatment, haemodialysis patients will experience various symptoms while focusing on coping with the disease's burden, reducing their ability to understand emotions. It is difficult to identify and express negative emotions, which contributes to the development of psychological disorders. Therefore, nursing staff should assist patients in identifying the source of symptom burden in a timely manner, focus on symptom distress relief, and actively guide MHD patients to achieve emotional relief, improve coping ability, and reduce depression levels.

Structural equation modeling results show that symptom burden predicts depression directly and indirectly through emotional distress, with the mediating effect accounting for 61.3% of the total effect. MHD patients devote significant energy to dealing with the disease and its symptoms throughout a lengthy treatment period. Most of them are unable to determine whether the source of their physical symptoms is due to their illness or to their psychological problems, which, combined with their difficulty identifying and expressing their emotions, exacerbates depression. Alexithymia is primarily associated with specific brain structures. Farah et al. (2018) demonstrated that the amygdala was a neurobiological factor associated with alexithymia and that an increase in the volume of the brain's right amygdala causes alexithymia. Changes in brain structure cause deliberate avoidance of emotional content, and reduced emotional activity causes atrophy of emotion-related brain regions, implying that brain structure changes are both a precursor and a result of related behaviors such as alexithymia (Förster et al., 2020). Cognitive impairment (CI) is one of the most common complications in MHD patients, with a prevalence of up to 69.7% (Tian et al., 2022). It is characterized by a deficit in one or more key brain functions, including memory, learning, decision-making, and attention. For MHD patients, it can result in a decrease in their ability to care for themselves and treatment adherence (Golenia et al., 2023). CIs are deficits in one or more key brain functions, such as memory, learning, decision-making, and attention that can impair MHD patients' ability to care for themselves and comply with treatment. They are also strongly linked to all-cause mortality, which significantly increases the burden on families and society. Previous studies (Carvalho et al., 2013; Helmes et al., 2008) have found that anemia, inflammation, and uremic toxins all impact CI in MHD patients. Currently, a large number of domestic and foreign studies on emotional dysfunction show that the incidence of emotional dysfunction is high, the occurrence of a wide range of groups, and emotional dysfunction will affect the individual's physical and mental health and social functioning, interfering with the treatment of the patient's existing illnesses and rehabilitation, and even aggravate the physical illnesses caused by the body's physical damage. This variable relationship also suggests that carers can not only improve patients' depression levels by alleviating the burden of symptoms but also focus on the multidimensional conditions of MHD patients' cognition and emotions by improving their ability to narrate their feelings, help patients improve their problems identifying their emotions through cognitive behavioral therapy (Yuan et al., 2003), and use supportive and expressive psychotherapies such as drawing, music, and other channels to enable patients to talk about their emotions freely and safely, break free from habitual problem-solving thinking, adopt new concepts and attitudes to look at old things, get out of a problematic situation, share their experiences, and promote mental health.

## 5 Conclusion

Patients undergoing MHD have a high symptom burden, which is significantly correlated with alexithymia and depression levels. This study found that alexithymia acts as a partial mediator in the relationship between symptom burden and depression in MHD patients, providing a new perspective on the complex interplay of emotional and physical health in this vulnerable population. By identifying and treating alexithymia, healthcare providers have the potential to improve patients' emotional awareness and psychological wellbeing, particularly in the management of depression symptoms. The findings indicate that interventions aimed at reducing alexithymia may be an effective way to alleviate the psychological distress associated with MHD. However, it is important to note that this study was carried out at a single location, which may limit the generalizability of the results. Future research should include multicenter studies with more diverse patient populations to validate and expand on these findings.

## 6 Limitations

This study is a single-center cross-sectional survey, which limits the findings' generalizability due to the small and unrepresentative sample size. The study is prone to selection and respondent bias, which could cause significant distortions in the results. As a result, the findings should be interpreted with caution. To address these limitations, in the future studies, multicenter designs with larger, more representative sample sizes can be used to improve the reliability and applicability of their findings. Additionally, longitudinal studies could shed light on the causal relationships between alexithymia, symptom burden, and depression in MHD patients.

## Data Availability

The original contributions presented in the study are included in the article/supplementary material, further inquiries can be directed to the corresponding author.
